# Climate and landscape context drive the functional composition of young tropical forests

**DOI:** 10.1002/ecy.70470

**Published:** 2026-09-04

**Authors:** Jazz J. M. Kok, Lourens Poorter, Maureen Nieuwschepen, Koen van Steenbeek, Tomonari Matsuo, Johan de Jong, Masha T. van der Sande, Lucy Amissah

**Affiliations:** ^1^ Forest Ecology and Forest Management Group Wageningen University & Research Wageningen The Netherlands; ^2^ Plant Ecology and Nature Conservation Group Wageningen University & Research Wageningen The Netherlands; ^3^ Laboratory of Geo‐Information Science and Remote Sensing Wageningen University & Research Wageningen The Netherlands; ^4^ CSIR‐Forestry Research Institute of Ghana Kumasi Ghana; ^5^ CSIR‐College of Science and Technology Accra Ghana; ^6^ Present address: Department of Urbanism, Faculty of Architecture and the Built Environment Delft University of Technology Delft The Netherlands

**Keywords:** climate, drought strategy, functional traits, Ghana, landscape context, secondary tropical forests

## Abstract

Human‐modified tropical landscapes are mosaics of farmland, young regrowing forests, and old‐growth forests. These fragmented landscapes are hotter and drier than forested landscapes and, combined with climate change, may lead to shifts in forest community composition. We asked how macroclimate and surrounding landscape shape community‐level drought strategies of young regrowing secondary forests. We assessed community composition of 37 young secondary dry and wet forests in Ghana during the first 3 years of succession, collected drought‐related leaf and stem traits of their pioneer species, and quantified surrounding landscape cover within an 800‐m radius around each plot. Macroclimate had the strongest influence on community drought strategies. Dry forest communities avoided drought through leaf shedding, delayed drought through small compound leaves that reduce heat load, and tolerated drought through dense wood and slow‐wilting leaves that both help maintain function during drought stress. Wet forest communities showed the opposite suite of traits, reflecting prioritization of fast growth under high water availability. Landscape context reinforced different forest recovery trajectories by further shaping community traits. Agricultural cover reduced community wood density in the dry forest, whereas it increased wood density in the wet forest and tended to increase drought traits such as small, compound, and deciduous leaves. Surrounding secondary forest cover promoted fast‐growing species with acquisitive leaves and low leaf mass per area in the dry forest, likely due to increased propagule pressure of pioneer species. Greater surrounding old‐growth cover increased leaf dry matter content in the wet forest, supporting the recruitment of conservative, shade‐tolerant late‐successional species. Macroclimate, landscape context, and their interaction thus jointly shape community functional composition and drought strategies of regrowing forests.

## INTRODUCTION

Human‐modified tropical landscapes consist of a mosaic of old‐growth forest (OGF), forests converted to agriculture and human settlements, and secondary forests that regrow on abandoned fields (FAO, 2022). Compared to OGFs, such landscapes are relatively open, hot, and dry, resulting in a more ruderal, drought‐adapted flora (Arroyo‐Rodríguez et al., 2023). Changes in landscape openness affect the local climate and the regional species pool and thus can affect the composition of regrowing secondary forests (Pinho et al., 2024) and the future of tropical forests in human‐modified landscapes. This may be further exacerbated by climate change, where an increased frequency and intensity of droughts may filter for a more drought‐tolerant community (McDowell et al., 2020; WMO, 2023). However, the composition of tropical forest communities does not change sufficiently fast to keep pace with climate change (Aguirre‐Gutiérrez et al., 2025), which may lead to increased mortality and shifts in ecosystem functioning. Understanding the drought adaptations of current forest communities is therefore crucial for designing restoration strategies that foster forests resilient to future climatic conditions. In this study, we compare two macroclimatic zones (i.e., dry and wet forest) as an analogy how wet forest may shift in species composition and community trait values in a drier future and assess how landscape context affects drought‐related traits in young secondary forests in these contrasting climates.

Tropical forests must cope with increased drought (WMO, 2023) and fragmentation (FAO, 2022). Prolonged seasonal droughts might favor hardier tree species and filter out those less able to tolerate drought stress (McDowell et al., 2020). Similarly, reduced landscape forest cover alters the pool of species available to recolonize cleared areas (Pinho et al., 2024) by favoring plants adapted to more exposed, hotter, and drier conditions and changes the abundance and composition of seed trees and animal dispersers (Arroyo‐Rodríguez et al., 2017; Dent & Estrada‐Villegas, 2021). Increasing drought and forest fragmentation may filter for drought‐adapted species and reduce species diversity as drought‐sensitive species disappear. It is important to understand how young secondary forests respond to climate and landscape changes because they are exposed to heat and drought, with little forest structure to buffer temperature or precipitation shifts (Schwartz et al., 2019). Early‐successional forest dynamics set the trajectory for long‐term forest development, making this stage a critical window for understanding future forest composition and guiding species selection for restoration that is future climate proof.

How young tropical forests respond to climatic and landscape changes can be explained by their functional trait composition. To survive drought stress, trees employ a suite of strategies that are determined by their root, stem, and leaf traits (Matheny et al., 2017). When resources are abundant, tree species prioritize growth, for example by developing large leaves to capture light and soft wood to transport water efficiently (Sterck et al., 2011, 2014). When resources are limited (e.g., under dry conditions), species prioritize survival, for example by producing smaller and tougher leaves that reduce water loss (Westoby, 1998) and dense wood that protects water‐transporting conduits from collapse (Sperry, 2003). These trait‐based strategies influence species' performance and determine their ability to establish and persist in a given environment (Violle et al., 2007). During forest succession, species must pass through multiple socio‐ecological filters such as resource availability, anthropogenic disturbance regimes such as fire and selective harvesting, and biotic interactions (Grime & Pierce, 2012; Poorter et al., 2023). As a result, the set of species that pass through these filters (by having traits that match local conditions) collectively shape community assembly (Keddy & Laughlin, 2021).

Within a landscape, forest cover shapes species availability and community assembly through two main mechanisms: microclimate and seed dispersal. A decline in forest cover creates more open conditions, which increase temperatures, intensify edge effects, and promote the dominance of early‐successional species. Forested areas offer cooler, more humid conditions that support species less adapted to drought, whereas agricultural lands expose vegetation to hotter, drier microclimates (Chen et al., 1999; Pinho et al., 2024). These conditions act as environmental filters, favoring the survival and dispersal of drought‐tolerant species and increasing their abundance in more open, agricultural landscapes (Baker et al., 2014).

At the same time, changes in forest cover influence the quantity and composition of seeds arriving at a site. Agricultural lands typically receive lower seed input and are dominated by early‐successional species dispersed by wind or generalist animals (Hordijk et al., 2024; Martínez‐Garza et al., 2011). In contrast, secondary forests provide more seed rain and a broader mix of early‐ and mid‐successional species, while OGFs supply the highest seed density and a larger proportion of late‐successional species. However, seed rain and the proportion of late‐successional species both decline with increasing distance from intact forest (Lawrence, 2004).

How the landscape affects community assembly may differ between dry and wet forests. In dry forests, where rainfall is lower (Engelbrecht et al., 2007), the influence of forest cover on community composition may be stronger, as forest patches can act as microclimatic refugia and sources of fast‐growing, drought‐sensitive species. In wetter forests, the contrast between forested and open areas is less pronounced, making it easier for less drought‐tolerant species to establish across the landscape. As a result, the influence of surrounding forest cover on the drought adaptations of regrowing communities is likely stronger in dry than in wet forest zones (Schönbeck et al., 2015).

While there is growing recognition that both macroclimate and landscape context shape forest communities, it remains unclear how these factors individually and jointly influence early secondary succession, particularly in terms of community‐level drought adaptations (Guariguata & Ostertag, 2001; Herrera‐Peraza et al., 2016). In this study, we investigate how macroclimate and landscape forest cover interact to shape the drought adaptation strategies of young secondary forests. We do so by assessing community composition for 37 young secondary dry and wet forests in Ghana, evaluating eight leaf and stem traits for their pioneer species, and quantifying surrounding landscape cover in an 800‐m radius around each plot as determined with remote sensing. We hypothesize that forests regrowing in drier climate zones, and in landscapes dominated by agriculture, will show a higher prevalence of drought‐adapted traits. Furthermore, we expect landscape effects to be more pronounced in dry forests than in wet forests, due to the stronger microclimatic contrast between forested and open areas in drier regions.

## METHODS

The study was conducted in sites in two tropical climate zones: a dry semi‐deciduous forest zone and a moist evergreen forest zone in Ghana (Appendix S1: Table S1, Figure S1; henceforth referred to as dry and wet). Although the dry and wet sites differ moderately in rainfall (1190 vs. 1808 mm/year), these forests differ strikingly in structure, phenology, and species composition (Hall & Swaine, 1976, 1981). The selected sites are part of the PANTROP research project that investigates the impacts of the landscape context on secondary forest succession on abandoned agricultural fields (Matsuo et al., 2023). We established 18 dry forest and 19 wet forest plots (25 m × 25 m) along a landscape forest cover gradient on fallow land that had been abandoned from agriculture and left to regrow naturally without further human intervention. In these plots, all woody plants (trees, shrubs, lianas) >1 cm stem dbh were tagged, identified, and measured for their dbh (1.3 m). Plots were established from 2020 to 2023, and plot age at the time of establishment varied from 0 years (i.e., immediately after land abandonment) to 2 years. The plots were located along a landscape forest cover gradient, ranging from two to 72% forest cover in the surrounding area in a radius of 800 m. The plots were monitored and measured annually until 2023 (*n* = 37 plots × 2–3 measurements = 78 plot‐year observations). This study, therefore, combines a chronosequence approach, comparing forests of different ages, with a longitudinal approach that tracks changes over time in the same plots. The dry forest plots contained 128 species, the wet forest plots 123 species, with 54 species in common. The species with the highest relative abundance at the dry site were *Chromolaena odorata* (33%), *Griffonia simplicifolia* (7%), and *Millettia zechiana* (6%), and at the wet site *Macaranga barteri* (21%), *Hurangana madagascariensis* (20%), and *Rauvolfia vomitoria* (10%; Appendix S1: Table S1). We collected landscape context data, forest community data, and eight functional traits as described below.

### Landscape context

To characterize the landscape context around each plot, we assessed the relative cover of agricultural land (crops, pasture, oil palm, rubber, and cocoa plantations), secondary forest (≤20 years), and OGF (>20 years). These landscape categories were selected because they represent a gradient of forest integrity and microclimatic conditions, from disturbed land with hot, dry conditions to developed forest with cool, humid conditions. They harbor distinct ecological niches, which influence regrowing forest communities by shaping dispersal patterns and species availability (Arroyo‐Rodríguez et al., 2017; Poorter et al., 2023).

Land use cover was quantified using a combination of Landsat satellite image time‐series and Sentinel‐2 land‐cover classification. The time‐series analysis was performed with the Anomaly Vegetation Change Detection (AVOCADO) time‐series algorithm (Decuyper et al., 2022). AVOCADO used all available Landsat images between 2000 and 2020 to identify forest cover and the age of newly established forest cover between 2000 and 2020. Land‐cover classification was performed with the RandomForest classifier in combination with cloud‐free Sentinel‐2 images in 2020, which groups pixels into objects based on spectral and textural information (de Jong et al., 2025; Tassi & Vizzari, 2020). Land cover was divided into 10 classes: (1) pioneer forest (<10‐year‐old forest), (2) young forest (≤20‐year‐old forest), (3) old forest (>20‐year‐old forest), (4) rubber plantation, (5) oil palm plantation, (6) cocoa plantation, (7) pasture/crops, (8) urban/bare soil, (9) water, and (10) other. The land‐cover maps were resampled to match the 30 m × 30 m pixels of the AVOCADO time‐series output. For analysis, pioneer and young forest were combined as *secondary forest*, and all cultivated classes (rubber, oil palm, cocoa, pasture/crops) were grouped as *agriculture*. A more detailed description of the remote sensing method can be found in de Jong et al. (2025).

Landscape effects on plot regeneration can vary across scales (Safar et al., 2022) because of differences in disperser movement ranges (Dent & Estrada‐Villegas, 2021), seed rain dynamics (Lawrence, 2004), and local environmental conditions (Baker et al., 2014). Therefore, landscape composition (% of each category) was calculated within increasing radii around the plot, ranging from 50 to 2000 m (50, 100, 200, 400, 800, 1200, 1600, 2000 m) around each plot. A zonal histogram was used to calculate the pixel count of each land use class within each buffer. The percentage cover for each category was calculated based on the total buffer area, adjusting for pixel overlap at the buffer edge (pixel size = 30 m × 30 m = 0.09 ha).

### Forest community data

#### Species selection

To quantify community‐level drought properties, we selected those species that collectively represented at least 80% of the total basal area per plot. A total of 59 species were studied with 36 species from the dry and 23 species from the wet forest. Species were sampled in young secondary forest adjacent to the plots to sample individuals growing under similar high light conditions and not to damage the plots. Only sun‐exposed individuals were included to compare species under optimal growing conditions where they can fully express their traits, except for certain species classified as shade‐bearers, which were found exclusively in the understory. Three to six trees (Arasa‐Gisbert et al., 2024) per species were selected that had a similar ontogenetic size range as trees in the forest plots with a dbh of 2–10 cm and a height of 2–8 m. For each tree, we determined six functional traits related to drought tolerance and resource acquisition: leaf turgor loss point (Ψ_tlp_), leaf mass per area (LMA, in grams per square centimeter), leaf dry matter content (LDMC, in milligrams per gram), leaf compoundness (LC, 0 = simple, 1 = compound), deciduousness (DE, 0 = evergreen, 1 = deciduous), and wood density (WD, milligrams per gram). Leaf turgor loss point was measured as described below. LMA, LDMC, LC, DEC, and WD data were obtained from earlier studies carried out at the same locations (Matsuo et al., 2023), which were measured following standardized protocols (Pérez‐Harguindeguy et al., 2013).

#### Leaf turgor loss point

Leaf turgor loss point (Ψ_tlp_) is the water potential threshold at which cells lose turgor pressure and leaves wilt (Bartlett et al., 2012). Ψ_tlp_ was measured from March to June 2021 and throughout 2023. To measure Ψ_tlp_, we sampled one healthy, fully expanded, sun‐exposed leaf from three to five individuals per species. Samples were collected either at sunrise and allowed to rehydrate for several hours or late in the afternoon for overnight rehydration. To rehydrate the samples, we recut the leaves underwater and placed them in buckets partially filled with water. The samples were covered with two polythene bags, which were sealed to block light and increase humidity, minimizing transpiration and optimizing rehydration. At the start of each measurement, we photographed the leaves to determine leaf area and measured their fresh mass to obtain turgid mass. We bench‐dried the leaves and measured their leaf mass and water potential repeatedly at short intervals until leaf mass stabilized. Afterward, the leaves were oven‐dried at 70°C for a minimum of 72 h to determine their dry mass. To determine Ψ_tlp_, we generated pressure‐volume (PV) curves (Sack et al., 2010) by measuring leaf water potential and corresponding water loss during desiccation. Water potential (Ψ) was measured using a pressure chamber (Model 1000 Pressure Chamber, PMS Instrument Company, Albany, USA), where increasing pressure forces water out of the leaf. Leaf volume was calculated from the mass of water lost at each pressure step. Ψ_tlp_ was identified as the breakpoint in the PV curve, where the relationship between pressure and water loss shifts from linear to nonlinear, which indicates the loss of turgor pressure. We used absolute Ψ_tlp_ (|Ψ_tlp_|) for further analysis with higher |Ψ_tlp_| values indicating greater leaf drought tolerance.

#### Community traits

We used community‐weighted means (CWMs) to characterize the dominant trait strategies within each forest plot. This approach captures the traits of the most abundant species, providing insight into which functional strategies are most strongly selected for by environmental filters such as macroclimate and landscape context. To describe the average trait value of the community, we calculated the CWM for each plot and census year by weighing the trait value of the species by their proportional basal area in the plot:

(1) $$\mathrm{CWM}=\sum_{i=1}^{S}w_{i}\times x_{i},$$

where *S* is the number of species making up at least 80% of total basal area, *w* is the relative basal area of species *i* and *x* is the trait value of a species. We weighted trait values by species' basal area rather than abundance, as this better reflects the ecological dominance and functional influence of species within the community. For species lacking trait data, we used genus‐level averages, which is appropriate given the strong trait similarity often observed among congeneric tropical tree species (Kattge et al., 2011).

### Statistical analysis

To select the most relevant landscape variables for statistical analyses, we explored landscape heterogeneity and the relationship between community traits and landscape context at different spatial scales using principal components analysis (PCA) using the “prcomp” function in R, and bivariate Pearson's correlation analyses using the “cor.test” function. The percentage coverage of each land use type was strongly correlated across all radii as well as between three groups of radii (0–800 m, 800–2000 m, and 0–2000 m), indicating that values of landscape metrics were consistent across these spatial scales. Relationships between community traits and landscape coverage of different land use types were also consistent across spatial scales. Based on this exploratory analysis, we selected the 0–800‐m radius for all three land use type coverages for subsequent analysis, as it is within the range of most plant species' dispersal distances (Corlett, 2009) while enhancing independency among plots by reducing overlap between radii.

We tested for multicollinearity among predictors in our linear models using the variance inflation factor (VIF), calculated with the “car” package in R (Fox et al., 2024). VIF values for the combinations of macroclimate, agriculture and secondary forest, and macroclimate, secondary forest and OGF, were close to 1, which indicates minimal collinearity (James et al., 2013). However, collinearity between other predictor combinations limited the number of predictors that could be included in a single model. Instead, we applied a hypothesis‐driven model selection approach, with each model designed to capture a different ecological mechanism (Table 1).

**TABLE 1:** Series of generalized linear mixed models to test the effects of macroclimate (dry/wet) and land‐cover types on eight community‐weighted mean (CWM) traits, and the underlying ecological mechanisms tested by each model.

Model	Response	Independent effect	Interaction effect	Ecological mechanism
1	CWM_trait_	Macroclimate	Not included	Climate as the dominant driver of community traits
2	CWM_trait_	Macroclimate + AG	Macroclimate × AG	Effects of landscape openness and microclimatic stress
3	CWM_trait_	Macroclimate + SF	Macroclimate × SF	Dispersal and establishment of pioneer species
4	CWM_trait_	Macroclimate + OGF	Macroclimate × OGF	Dispersal and establishment of late‐successional species
5	CWM_trait_	Macroclimate + AG + SF	Macroclimate × AG; Macroclimate × SF	Combined effects of openness and pioneer colonization
6	CWM_trait_	Macroclimate + SF + OGF	Macroclimate × SF; Macroclimate × OGF	Combined effects of pioneer and late‐successional colonization

*Note*: Models included macroclimate (dry vs. wet) and one or two land‐cover predictors, with interaction terms evaluated when applicable. Plot identity was included as a random effect to account for repeated measurements.

Abbreviations: AG, agriculture; OGF, old‐growth forest; SF, secondary forest.

To evaluate the effects of macroclimate and forest cover on community‐mean traits while accounting for non‐normal trait distributions, we applied a series of generalized linear mixed models (GLMMs; Table 1) using the “glmmTMB” package (Brooks et al., 2017). Each community trait (WD_CWM_, |Ψ_tlp_|_CWM_, DE_CWM_, LC_CWM_, LMA_CWM_, LDMC_CWM_, LA_leaflet CWM_, LA_total CWM_) was modeled as a response variable, with macroclimate (dry/wet) and land‐cover types (% agriculture, secondary forest, OGF cover) as fixed effects. Interaction terms were included to test whether the effect of land‐cover types differed between macroclimates. Plot identity was included as a random effect to account for repeated measurements.

Model selection was conducted using the dredge function in the MuMIn package (Bartoń, 2025) to identify top‐ranked models based on difference in Akaike information criterion between models (ΔAIC < 2). Because agricultural and OGF cover were collinear, we excluded models containing both predictors a priori by constraining the dredge function. When several models had a ΔAIC < 2 compared with the best fitting model, we used model averaging to obtain weighted parameter estimates and relative variable importance (VI). Averaging was performed with the model.avg. function in the MuMIn package, which calculates a weighted mean of parameter estimates across the selected models based on each model's Akaike weight (i.e., models with lower AIC values contribute more to the averaged estimates). Model‐averaged coefficients represent unconditional effect estimates across all plausible models where the predictor was included (Burnham & Anderson, 2002). Marginal and conditional *R* ^2^ values were obtained from the top‐ranked GLMM (lowest AIC_c_). To test for macroclimate differences, estimated marginal means from each trait model were extracted using the “emmeans” package (Lenth et al., 2025) and pairwise comparisons between dry and wet macroclimates were performed. While landscape composition differed between macroclimatic zones (dry forest plots tended to have higher agricultural cover (AG), whereas wet forest plots were more often surrounded by OGF; Appendix S1: Figure S2), this modeling approach allowed us to test for the independent and interactive effects of macroclimate and landscape context on community traits.

All data analyses were performed using R version 4.4.2 (R Core Team, 2024).

## RESULTS

**FIGURE 1:**
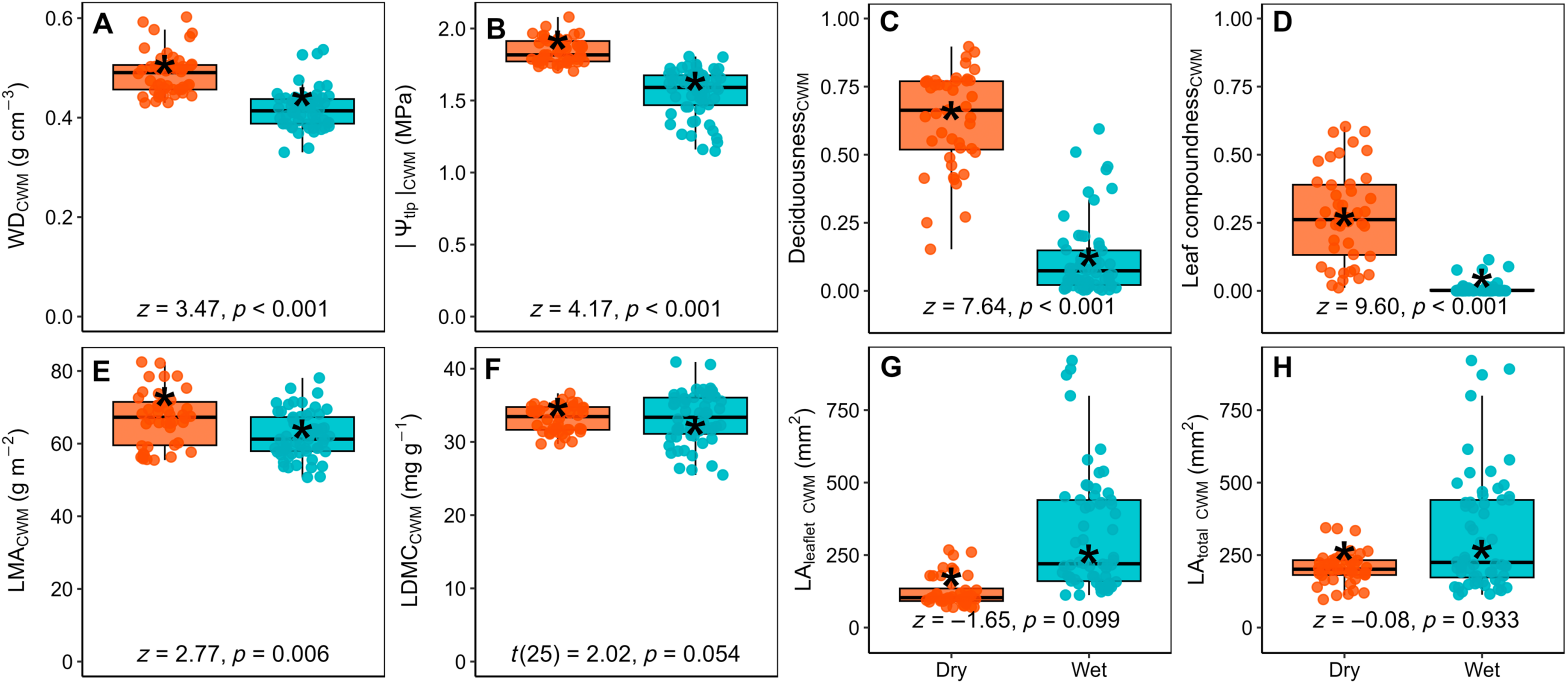
Boxplots of eight community traits in dry (red, *n* = 42) and wet (blue, *n* = 36) forests. The lower, middle (thick line), and upper boundaries indicate the 25th, 50th, and 75th percentiles. Dots represent individual plots; asterisks denote the estimated marginal means (EMMs) derived from (generalized) linear mixed models accounting for covariates and random effects. For the pairwise comparisons of EMMs, *p*‐values for generalized linear mixed model (GLMM) traits (Gamma family with log link) are based on Wald *z*‐tests (df = ∞), whereas *p*‐values for leaf dry matter content (LDMC) (Gaussian LMM) are based on *t*‐tests with Satterthwaite degrees of freedom. |Ψ_tlp_|, absolute leaf turgor loss point; CWM, community‐weighted mean; LA_leaflet_, leaflet area; LA_total_, total leaf area; LDMC, leaf dry matter content; LMA, leaf mass per area; WD, wood density.

We used GLMMs to evaluate how community drought adaptations of early secondary forest are impacted by macroclimate, surrounding landscape, and their interaction. Dry forest were, compared to wet forests, dominated by species with significantly higher WD, more negative (i.e., higher absolute) turgor loss point values, a greater proportion of deciduous and compound‐leaved species, higher mean LMA and LDMC, and smaller leaflet area (Figure 1A–G). Total leaf area did not differ between forest types (Figure 1H), likely because compound‐leaved species in dry forests maintain overall leaf area through multiple smaller leaflets, which is an adaptation linked to convective heat loss in dry, open environments (Xu et al., 2009). Macroclimate had the strongest and most significant effect (i.e., traits were significantly different in wet forests compared to dry forests, which were the reference level in our models) for seven out of eight traits (Table 2), whereas surrounding landscape and its interaction with macroclimate had a significant effect on four out of eight traits. The variation explained by the fixed effects (climate, landscape cover, and their interactions) was on average 56% and ranged from 27% for LDMC to 85% for LC.

**TABLE 2:** Results of (generalized) linear mixed models testing the effects of macroclimate and surrounding land‐cover types on eight community‐weighted mean traits.

CWM_trait_ and model statistics	(Intercept)	M	AG	M × AG	SF	M × SF	OGF	M × OGF	*R* ^2^_fixed_	*R* ^2^_random_ (plot ID)
WD										
*β*	**−0.72**	**−0.13**	−0.04	**0.10**	…	…	0.02	**−0.12**	0.59	0.25
SE	**±0.04**	**±0.03**	±0.03	**±0.04**	…	…	±0.05	**±0.05**		
*p*	**<0.001**	**0.015**	0.204	**0.025**	…	…	0.653	**0.037**		
VI	**NA**	**1**	0.30	**0.30**	…	…	0.40	**0.27**		
|Ψ_tlp_|										
*β*	**0.60**	**−0.16**	0.01	…	…	…	−0.03	…	0.55	0.20
SE	**±0.03**	**±0.05**	±0.02	…	…	…	±0.03	…		
*p*	**<0.001**	**0.001**	0.550	…	…	…	0.230	…		
VI	**NA**	**1**	0.19	…	…	…	0.32	…		
DE										
*β*	**−0.54**	**−1.93**	0.24	0.25	…	…	**−0.31**	…	0.76	0.13
SE	**±0.21**	**±0.35**	**±**0.16	±0.26	…	…	**±0.18**	…		
*p*	**0.013**	**<0.001**	0.144	0.362	…	…	**0.010**	…		
VI	**NA**	**1**	0.58	0.19	…	…	**0.22**	…		
LC										
*β*	**−1.56**	**−4.23**	0.38	0.65	−0.16	…	−0.36	−0.86	0.85	0.11
SE	**±0.37**	**±0.63**	±0.30	±0.45	±0.25	**…**	±0.44	±0.61		
*p*	**<0.001**	**<0.001**	0.213	0.156	0.531	**…**	0.420	0.165		
VI	**NA**	1	0.62	0.23	0.11	**…**	0.27	0.12		
LMA										
*β*	**4.22**	**−0.12**	**…**	**…**	**−0.08**	**0.10**	**…**	**…**	0.46	0.34
SE	**±0.04**	**±0.06**	**…**	**…**	**±0.05**	**±0.05**	**…**	**…**		
*p*	**<0.001**	**0.036**	**…**	**…**	**0.090**	**0.060**	**…**	**…**		
VI	**NA**	1	**…**	**…**	**0.72**	**0.44**	**…**	**…**		
LDMC^s^										
*β*	**33.1**	**−2.47**	**…**	**…**	**…**	**…**	−0.02	**2.93**	0.27	0.60
SE	**0.75**	**1.13**	**…**	**…**	**…**	**…**	±0.82	**±1.13**		
*p*	**<0.001**	**0.038**	**…**	**…**	**…**	**…**	0.979	**0.015**		
VI	NA	NA	**…**	**…**	**…**	**…**	NA	NA		
LA_leaflet_										
*β*	**4.81**	**0.61**	−0.19	**…**	0.17	**…**	0.26	0.26	0.66	0.24
SE	**±0.19**	**±0.33**	±0.12	**…**	±0.11	**…**	±0.17	±0.28		
*p*	**<0.001**	**0.051**	0.107	**…**	0.133	**…**	0.126	0.436		
VI	**NA**	1	0.12	**…**	0.42	**…**	0.69	0.20		
LA_total_										
*β*	**5.32**	0.20	−0.06	−0.34	0.11	**…**	0.19	0.29	0.33	0.47
SE	**±0.17**	±0.17	±0.15	±0.21	±0.11	**…**	±0.17	±0.27		
*p*	**<0.001**	0.496	0.693	0.109	0.342	**…**	0.265	0.285		
VI	**NA**	1	0.27	0.14	0.20	**…**	0.45	0.12		

*Note*: Model *R* ^2^ values are based on the top‐ranked model with the lowest Akaike information criterion (AIC). Bold parameter estimates are (near) significant, and ellipses (…) indicate terms excluded from the model. For climate effects, dry forest was used as the reference level. Model outputs are based on conditional model averaging unless otherwise indicated; *s* denotes output from a single top model, and NA indicates not applicable.

Abbreviations: |Ψ_tlp_|, absolute leaf turgor loss point; *β*, standardized regression coefficient; AG, agriculture; CWM, community‐weighted mean; DE, proportion of deciduous species; LA_leaflet_, leaflet area; LA_total_, total leaf area; LC, proportion of species with compound leaves; LDMC, leaf dry matter content; LMA, leaf mass per area; M, macroclimate; OGF, old‐growth forest; *p*, significance level; Plot ID, plot identity; SE, standard error; SF, secondary forest; VI, relative variable importance; WD, wood density.

**FIGURE 2:**
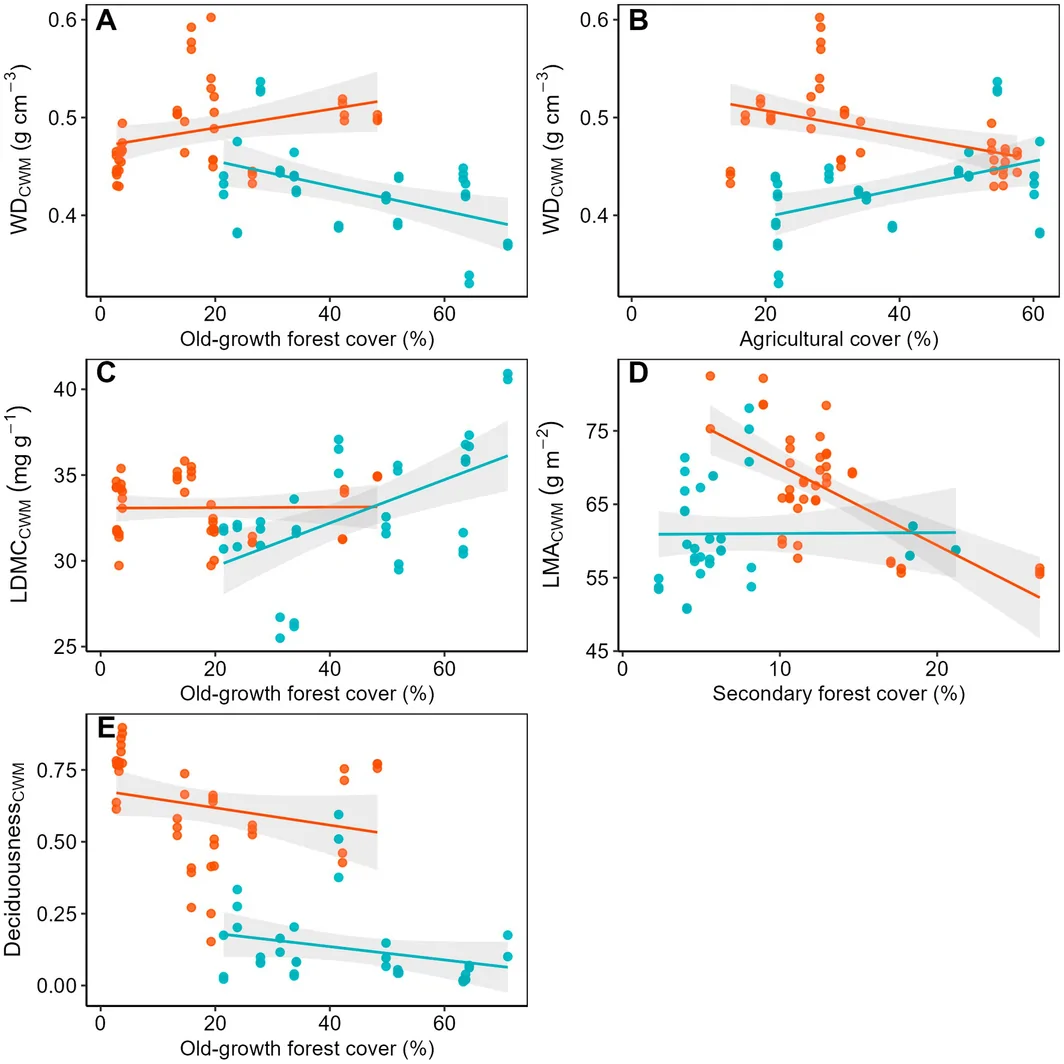
Site‐specific relationships between surrounding land use (% cover within an 800‐m radius) and community‐weighted mean (CWM) trait values for the five traits for which the landscape context was included in the best fitting (generalized) linear mixed model. Points represent individual forest plots (*n* = 37 plots × 2–3 measurements = 78 plot‐year observations), and linear regression lines with 95% CIs are shown for each macroclimatic zone (dry forest in orange, wet forest in blue). Panels show separate traits; the *y*‐axis indicates trait values (with units where applicable), and the *x*‐axis shows the relevant land‐cover variable from the selected model (agriculture, secondary forest, or old‐growth forest). Trend lines were fitted using linear models (geom_smooth(method = “lm”)) and are shown for visualization only; statistical inference is based on the full model reported in Table 2. For model details, see Table 2. LDMC, leaf dry matter content; LMA, leaf mass per area; WD, wood density.

The surrounding landscape affected structural and phenological drought‐related adaptations, but its effect varied with traits and macroclimate. Surrounding AG decreased WD in dry forest and increased community WD in the wet forest (Table 2; Figure 2B). Agriculture also increased DE, LC, LA_leaflet_, and LA_total_ but nonsignificantly. In the dry forest, surrounding secondary forest (SF) cover decreased community LMA (Figure 2D), while in the wet forest surrounding OGF cover increased community LDMC (Figure 2C). OGF had a near‐significant negative effect on the proportion of deciduous species in both dry and wet forests (Table 2, Figure 2E).

## DISCUSSION

We asked how macroclimate and landscape context affect drought adaptations in dry and wet secondary tropical forests. Macroclimate was the dominant driver of community strategies, with dry forests being more adapted to drought than wet forests. Within each forest type, landscape context further influenced these patterns: Agricultural landscapes decreased WD in the dry forests but increased WD in wet forests and tended to favor leaf drought‐adapted traits across forests; in dry forests, surrounding secondary forests promoted soft, acquisitive leaves that enhance growth; and in wet forests, surrounding OGFs increased leaf toughness, which enhances shade tolerance. These findings suggest that macroclimate shapes overall drought strategies, while landscape context further reinforced the functional composition of regrowing communities.

### Dry forest communities avoid, delay, or tolerate drought

Dry forest species cope with drought through three main strategies: avoidance, delay, or tolerance (Poorter et al., 2021). Accordingly, we found that dry forest communities avoid water loss through a drought‐deciduous leaf habit (Figure 1C; cf. Zhao et al., 2022). They delay drought effects by having compound leaves with small leaflets (Figure 1G), which reduce overheating and transpiration due to a thin boundary layer that facilitates convective cooling (Eker et al., 2022; Xu et al., 2009). Furthermore, dry forest communities tolerate drought by producing tough leaves with high LMA and LDMC (Figure 1E,F), which are associated with thick cell walls, low water content, and a high modulus of elasticity, which allow leaves to remain rigid under drought stress and contribute to a higher |Ψ_tlp_| (Figure 1B; Niinemets, 2001). Additionally, higher WD increases drought tolerance of dry communities by reinforcing stems with dense structural tissues that prevent collapse of cavitated vessels (Figure 1A; Hacke et al., 2006; Markesteijn et al., 2011). These adaptations collectively reduce water loss and maintain functionality during drought periods (Bartlett et al., 2012; Poorter et al., 2010).

In contrast, wet forest communities showed the opposite suite of traits. With greater water availability, they prioritize fast growth and higher transpiration rates to compete for light (Sterck et al., 2006). Wet communities had larger, simple leaves (Figure 1D,H; Wright et al., 2017) that enhance light interception, and softer leaves with lower LMA (Figure 1E), which facilitates rapid biomass accumulation. Matsuo et al. (2023) evaluated leaf traits and strategies of 122 early‐successional Ghanian tree species from dry and wet forests. Similarly, they found that dry species were more likely to avoid and delay drought by having a deciduous leaf habit and compound leaves. In contrast to our findings, they found that wet species had a higher LMA and LDMC than dry species, which may reflect greater shade tolerance. The results may be different because they included more species (122 vs. 58 in our study) thus also sampling more evergreen shade‐tolerant species, and because the dominant species that successfully passed the environmental filters may differ more strikingly in their trait values than subordinate species. These differences indicate that species level differences do not necessarily scale up to community‐level differences.

Overall, climate strongly drives all community drought adaptations in young forests, with early‐successional dry forest communities consistently showing stronger drought adaptations than wet forest communities.

### Agricultural landscapes tend to favor drought‐adaptive traits

We expected that greater surrounding agricultural land cover would increase the abundance of drought‐adapted species in the landscape, thereby enhancing community‐level drought adaptations in young regrowing forests. Agricultural land is more open and characterized by higher temperatures and lower humidity compared to forests (Arroyo‐Rodríguez et al., 2017), which favors drought‐delaying species. Indeed, we found that in both macroclimates, greater surrounding agricultural area tended to increase several drought traits such as small, compound, and deciduous leaves (Table 2). This indicates that agricultural landscapes filter for drought‐delaying traits, such as smaller leaves that minimize transpirational water loss (Wright et al., 2017). In wet forests, higher AG also increased the abundance of compound‐leaved species (Table 2). Compound leaves tend to have small leaflets with thin boundary layers that enhance convective heat loss (Gates, 1968), and some species exhibit photonastic leaf movement that reduces water loss under high irradiance.

Additionally, AG significantly affected WD, and this effect contrasted between macroclimates. In the dry forest, WD tended to decline with increasing AG (Table 2, Figure 2B), possibly because more open, agricultural landscapes favor lighter wooded, fast‐growing species that are wind dispersed and may colonize early‐successional sites more easily. In contrast, in wet forests agriculture‐dominated landscapes resulted in communities dominated by denser wooded species (Figure 2B) perhaps because these landscapes have been used more intensively and have experienced more shifting cultivating cycles. Repeated slashing and burning filters for dense‐wooded species that have a high resprouting ability (Jakovac et al., 2016; Poorter et al., 2010). Additionally, more human‐modified landscapes (i.e., those with higher agricultural or secondary forest cover) experience greater human activity, which can further affect propagule availability and impose landscape‐level legacy effects that favor specific species during early succession.

The capacity of young forests to adapt to different climatic and landscape contexts suggests a promising degree of resilience. However, this adaptation may come at the cost of functional and taxonomic diversity, as drought‐sensitive and late‐successional species may be filtered out in agricultural areas. As a result, reduced response diversity can increase vulnerability to additional stressors such as pests or disease. Moreover, open, fragmented landscapes expose regenerating forests to more extreme drought conditions compared to more buffered, forested areas, potentially pushing even drought‐tolerant species beyond their limits. Therefore, while young forests show adaptive potential in dry and human‐modified environments, this resilience is not without ecological trade‐offs.

### Secondary forest landscapes favor acquisitive leaves

Similar to our expectation that forested landscapes reduce drought adaptations, we found that surrounding secondary forest cover decreased community‐weighted LMA (Table 2). Additionally, in the dry forest, higher surrounding secondary forest cover was negatively correlated with community‐weighted |Ψ_tlp_| (Appendix S1: Figure S3A). These patterns likely reflect an increased dominance of pioneer species, which may result from facilitated establishment and enhanced dispersal from surrounding secondary forests. Secondary forests can create more favorable microclimatic conditions (i.e., lower irradiance, cooler temperatures, and higher humidity), which reduce water stress and increase light competition (Guariguata & Ostertag, 2001). This favors the establishment of fast‐growing species with larger, thinner, and softer leaves (Hassiotou et al., 2010; Lohbeck et al., 2015), but reduces drought tolerance (Niinemets, 2001). In contrast, open landscapes expose seedlings to harsher microclimates, which may filter for more drought‐tolerant species (Arroyo‐Rodríguez et al., 2017). Furthermore, secondary forests likely enhance seed availability and dispersal by serving as sources of pioneer species and increasing seed rain into adjacent regrowing areas (Arroyo‐Rodríguez et al., 2017; Mesquita et al., 2001). Although many studies assume this, few studies have actually tested this.

In line with our hypothesis, surrounding secondary forest cover appeared to influence community traits more strongly in the dry forest. Similar to |Ψ_tlp_|, the effect of secondary forest on LMA was primarily driven by the dry forest, while wet forests showed no consistent response (Figure 2D; Appendix S1: Figure S3a,b). This absence of an effect in the wet forest may reflect a true ecological pattern under more benign climatic conditions, but could also be influenced by limited variation in surrounding secondary forest cover (Appendix S1: Figure S2), which may reduce our ability to detect trait responses. Nevertheless, in the dry forest, lower rainfall and more variable conditions likely strengthen environmental filtering of community traits, especially in young forests with limited microclimatic buffering due to less canopy development. Accordingly, the functional composition of secondary forests may differ more strongly from that of OGFs in the dry region than in the wet region (Figure 1B,E). These stronger trait contrasts, combined with a harsher climate, may enhance the influence of secondary forest cover on regenerating communities in the dry forest. In contrast, the more benign and stable conditions in the wet forest may reduce both environmental filtering and functional differences between secondary and OGFs, dampening the effect of secondary forest cover on the trait composition of regenerating communities.

Hence, surrounding secondary forest cover favors the establishment of fast‐growing, more drought‐sensitive species, particularly in dry forests where colonization from surrounding vegetation may play a stronger role in shaping community functional composition.

### Old‐growth forest landscapes favor tough leaves

Contrary to our expectation, high surrounding OGF cover (>20 years) was associated with higher community LDMC of young secondary forest in wet forests, but not in dry forests. OGFs are typically dominated by late‐successional, shade‐adapted species that invest in structurally reinforced leaves with high dry matter content, which enhances leaf lifespan (Kitajima & Poorter, 2010; Reich et al., 1995) and extends the photosynthetic revenue stream under low light conditions (Sterck et al., 2006). Proximity to OGF may increase seed rain of such species into regenerating areas, accelerating the successional shift toward higher community LDMC.

In contrast, LDMC did not respond to OGF cover in the dry forest. This likely reflects the dominance of drought‐deciduous species and the prevalence of compound leaves in dry forest communities (Figure 1C,D), resulting in less variation in community LDMC (range LDMC_dry_ = 29.7–36.6 mg g^−1^; range LDMC_wet_ = 25.5–40.9 mg g^−1^). Although OGF cover tended to be lower in the dry forest (Appendix S1: Figure S2), landscape variation was sufficient to detect responses in other traits (Appendix S1: Figure S3A). This supports the interpretation that the absence of a response for LDMC is more likely due to ecological constraints (such as trait convergence under drought) than to limited variation in landscape context.

Thus, surrounding OGF cover promotes communities that invest more in leaf tissue durability in wet forests, while in dry forests, trait convergence may constrain such functional responses to the landscape.

## CONCLUSIONS

Our study demonstrates how macroclimate and surrounding landscape context jointly shape drought strategies in young tropical forests regrowing after agricultural abandonment. Macroclimate emerged as the primary driver, with dry forests showing stronger drought adaptations than wet forests. Landscape context reinforced different forest recovery trajectories by further shaping community functional composition in climate‐specific ways. These findings highlight the importance of considering both regional climate and landscape context when predicting or guiding forest regeneration under changing environmental conditions. In dry forests, surrounding secondary forest cover can support the regeneration of fast‐growing, acquisitive species that might otherwise be lost in agricultural landscapes, where communities are filtered toward more drought‐tolerant traits. In wet forests, OGF may promote the establishment of more conservative species that are less likely to regenerate in fragmented or agriculture‐dominated areas. Maintaining or restoring forest patches across the landscape can enhance seed arrival and help shape the functional composition of forest regrowth.

## CONFLICT OF INTEREST STATEMENT

The authors declare no conflicts of interest.

## Supporting information


Appendix S1.

## Data Availability

Data and code (Kok, 2026) are available in the Data Archiving and Networked Services (DANS) repository at https://doi.org/10.17026/LS/VGRHK2.
