# Supplementary material for: Climate and landscape context drive the functional composition of young tropical forests
Source: Ecology. 2026 Sep 4;107(9):e70470. doi: 10.1002/ecy.70470 (PMC13543193; doi:10.1002/ecy.70470)
Supplement: Supplementary file 1 [file ECY-107-e70470-s001.pdf]

Appendix S1

Climate and landscape context drive the functional composition of young tropical forests

Jazz J.M. Kok, Lourens Poorter, Maureen Nieuwschepen, Koen van Steenbeek, Tomonari Matsuo, Johan de Jong, Masha T. van der Sande, Lucy Amissah

Ecology

Table S1. Characteristics of the dry and wet forest sites in Ghana, including plot characteristics, location, climate, and forest characteristics.

**Site characteristic**	**Dry forest**	**Wet forest**
Number of plots	18	19
Size plot	25 × 25 m	25 × 25 m
Location		
– Latitude	7°11' N, 1°73' W	5°10' N, 2°02' W
– Community, region	Abofour, Ashanti region	Bonsa, Western region
Mean annual rainfall	1290 mm[2]	1808 mm[2]
Mean monthly temperature		
– Maximum	30.6°C[3]	32.0°C[3]
– Minimum	21.2°C[3]	22.8°C[3]
Dry season		
– Months	Nov/Dec–Feb[4]	Nov/Dec–Feb[4]
– Mean monthly rainfall	28 mm[3]	82.6 mm[3]
Forest type	Dry semideciduous[1]	Wet/moist evergreen[1]
Species composition (3-year-old secondary forest)		
– # species across plots	128	123
– # shared species across forests	54	54
– Top 10 dominant species across plots with relative abundance (%) and shade tolerance guild[5]	*Chromolaena odorata* (33%; SLP); *Griffonia simplicifolia* (7%; LLP); *Millettia zechiana* (6%; SLP); *Broussonetia papyrifera* (6%; SLP); *Antiaris toxicaria* (5%; LLP); *Ficus exasperata* (5%; SLP); *Milletia rhodanta* (4%; SLP); *Baphia nitida* (4%; PST); *Albizia zygia* (3%; LLP); *Solanum erianthum* (2%; SLP)	*Macaranga barteri* (21%; SLP); *Harungana madagascariensis* (20%; SLP); *Rauvolfia vomitoria* (10%; SLP); *Alchornea cordifolia* (10%; SLP); *Macaranga hurifolia* (8%; SLP); *Ficus sur* (6%; SLP); *Ficus exasperata* (5%; SLP); *Musanga cecropioides* (3%; SLP); *Tetrorchidium didymostemon* (2%; SLP); *Anthocleista nobilis* (2%; SLP)

[1] Hall & Swaine (1976), [2] Ghana Meteorological Service records (data 1973-2009), [3] Amissah et al. (2018), [4] Matsuo et al. (2023), [5] Finegan (1996)

Abbreviations: SLP = short-lived pioneer; LLP = long-lived pioneer; PST = partial shade tolerant.

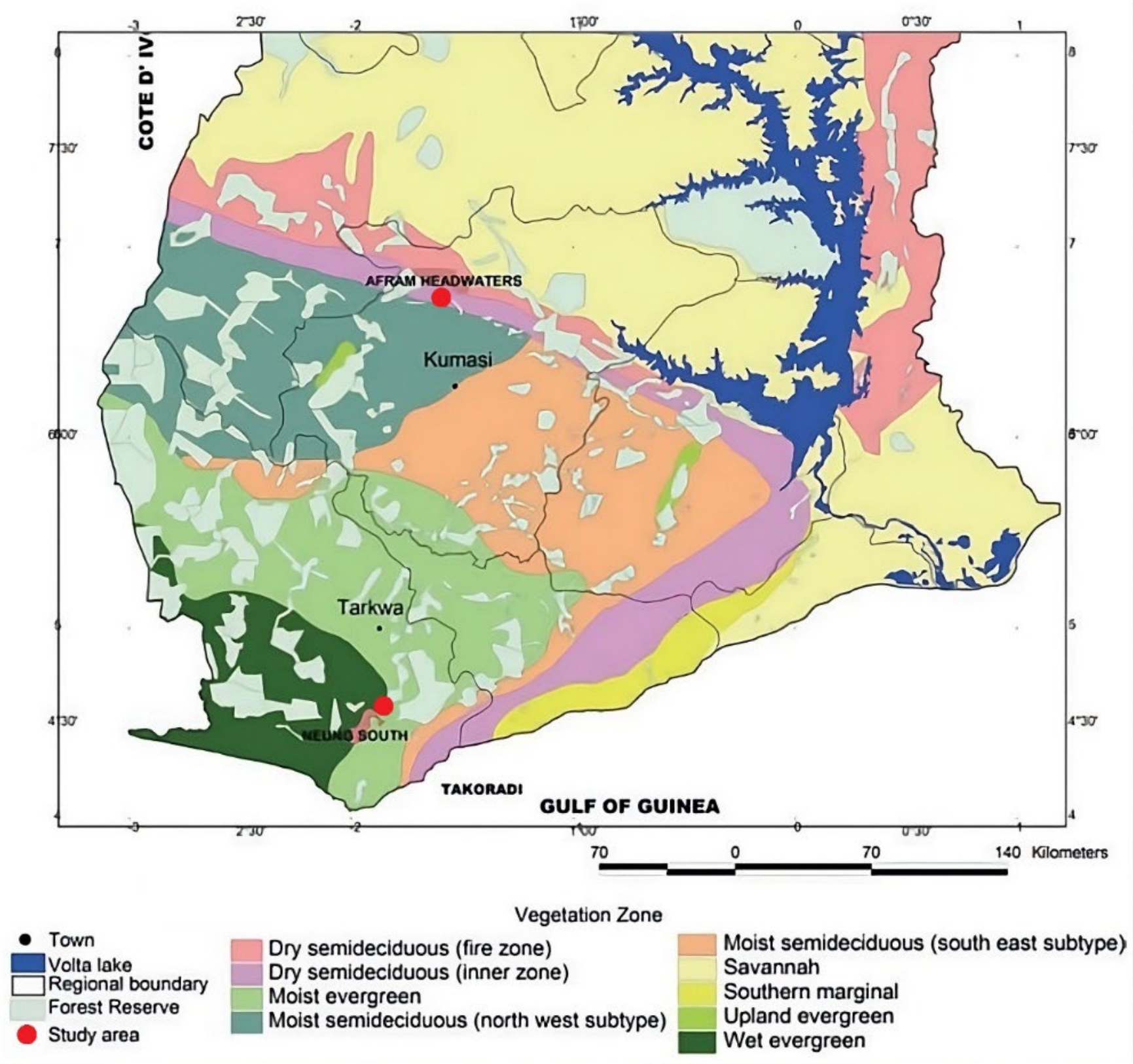

Figure S1. Map showing the vegetation zones of Ghana, forest reserves and the study sites Abofour (dry forest site near Afram Headwaters Forest Reserve) and Bonsa (wet forest site near Neung South Forest Reserve). Adapted from: Amissah (2014).

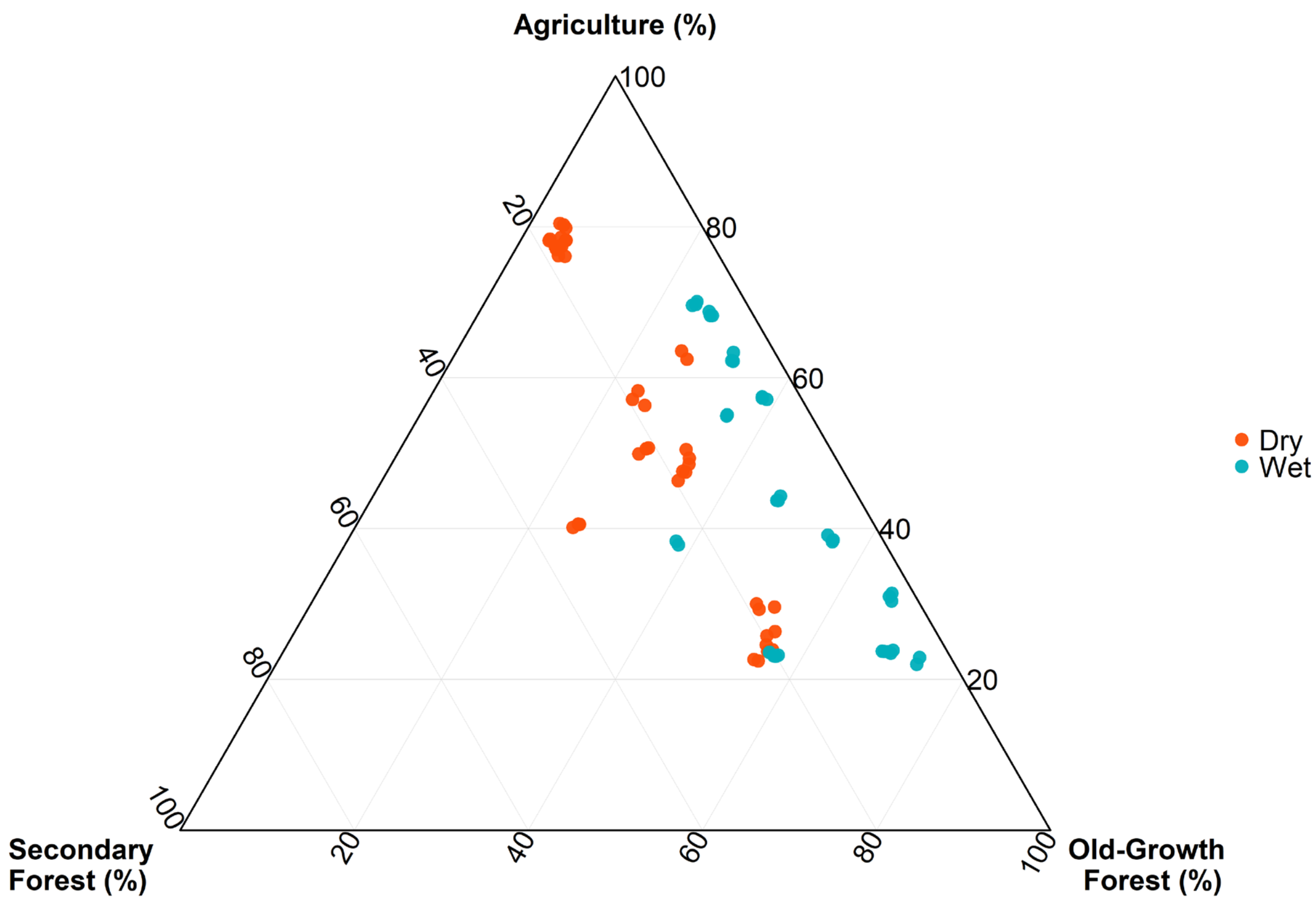

Figure S2. Ternary plot showing the percentage of surrounding land cover (agriculture, secondary forest, and old-growth forest) within an 800 m radius of each plot. Each point represents the census of a plot (n = 37 plots × 2–3 measurements = 78 plot-year observations), colored by macroclimate (orange = dry; blue = wet).

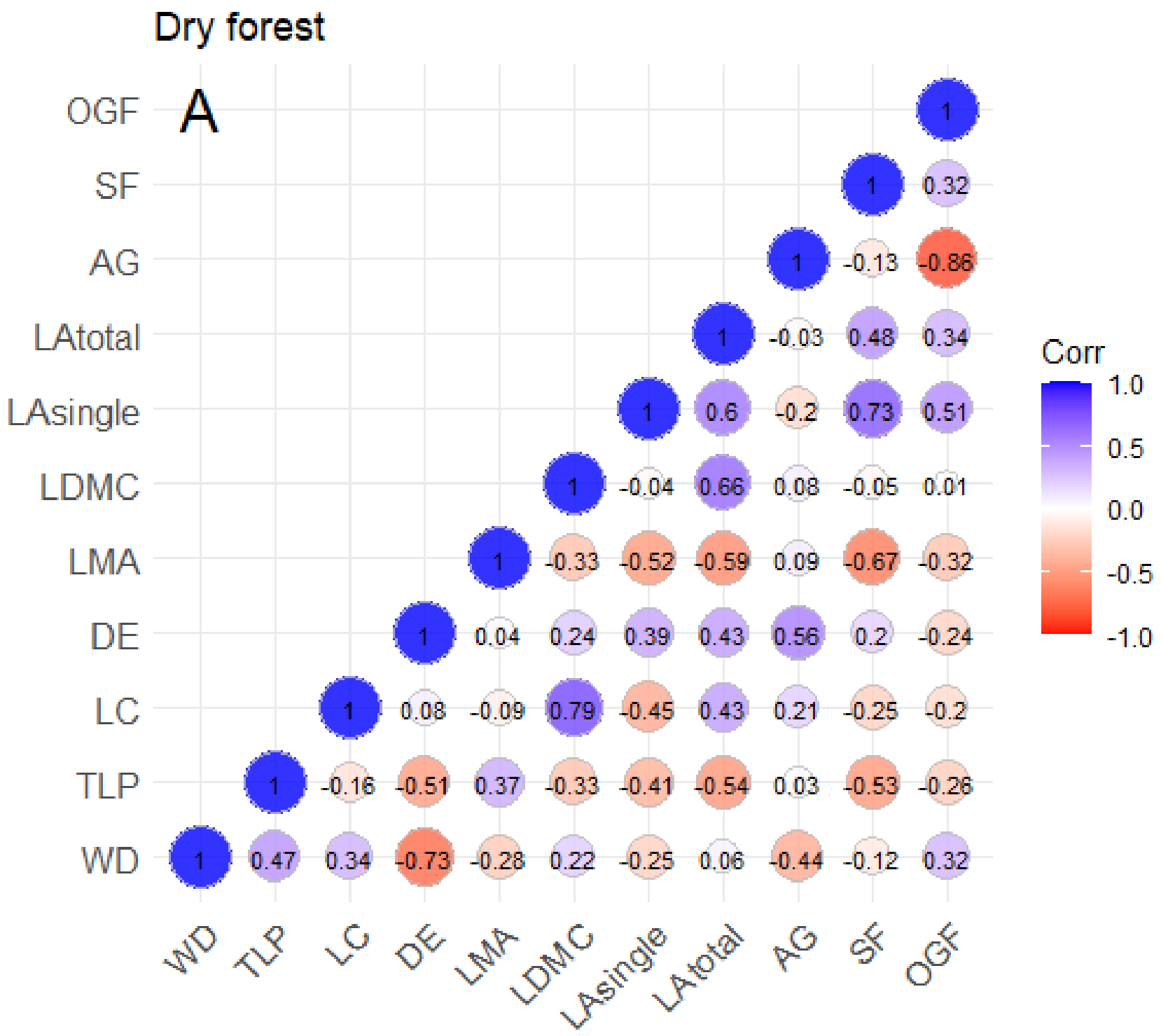
Dry forest
A
OGF
SF
AG
LAtotal
LAsingle
LDMC
LMA
DE
LC
TLP
WD
Corr
1.0
0.5
0.0
-0.5
-1.0

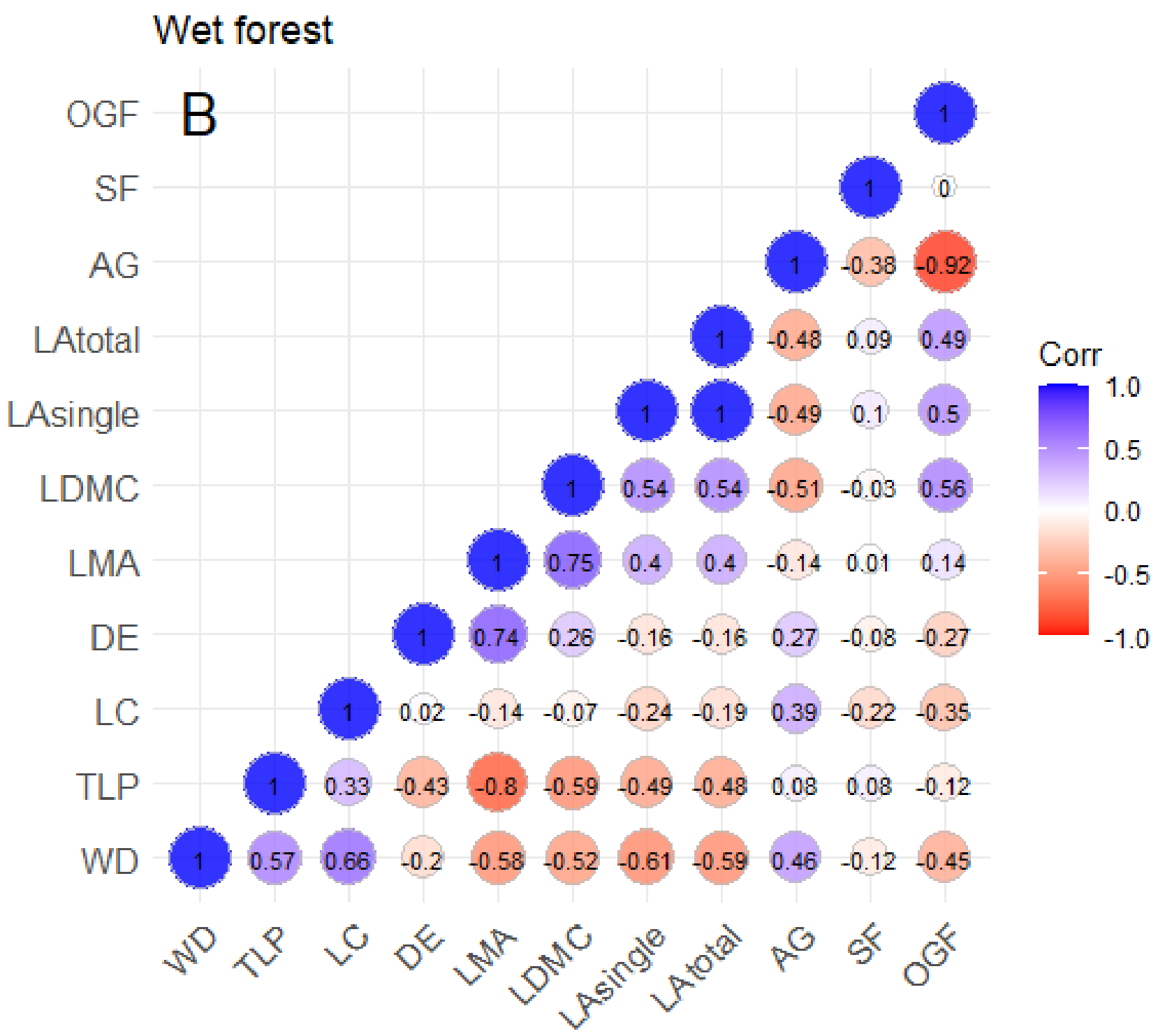
Wet forest
B
OGF
SF
AG
LAtotal
LAsingle
LDMC
LMA
DE
LC
TLP
WD
Corr
1.0
0.5
0.0
-0.5
-1.0

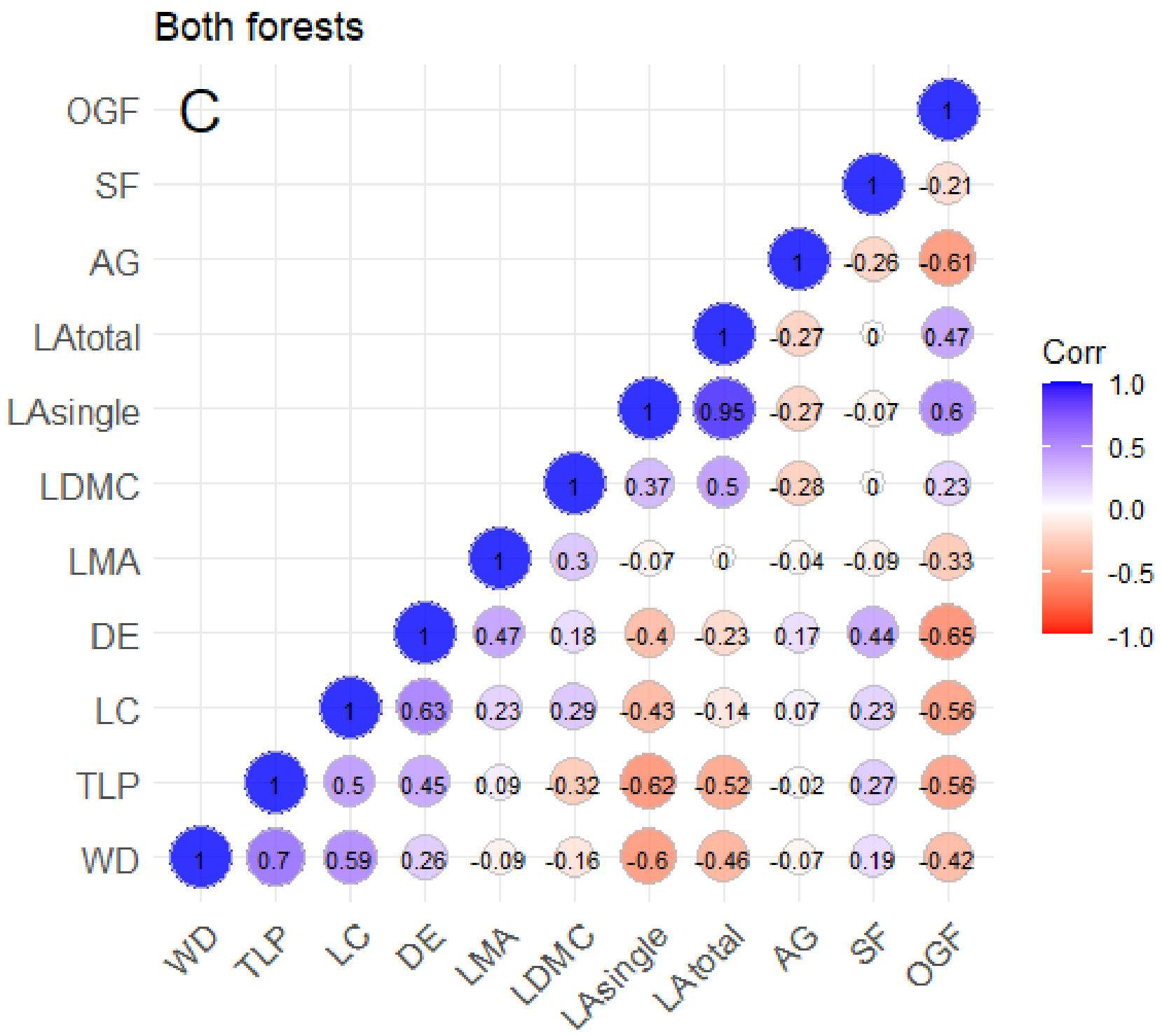

Figure S3. Pearson's r correlation matrices showing the bivariate relationships between eight community traits and three land cover classes (agriculture, secondary forest, old-growth forest) in (A) dry forest, (B) wet forest, and (C) both forests.

References

Amissah, L. (2014). Functional traits, drought performance, and the distribution of tree species in tropical forests of Ghana. Doctoral dissertation, Wageningen University.

Amissah, L., Mohren, G. M. J., Kyereh, B., Agyeman, V. K., & Poorter, L. (2018). Rainfall seasonality and drought performance shape the distribution of tropical tree species in Ghana. Ecology and Evolution, 8(16), 8582–8597. https://doi.org/10.1002/ece3.4384

Finegan, B. (1996). Pattern and process in neotropical secondary rain forests: The first 100 years of succession. Trends in Ecology & Evolution, 11(3), 119–124. https://doi.org/10.1016/0169-5347(96)81090-1

Hall, J. B., & Swaine, M. D. (1976). Classification and Ecology of Closed-Canopy Forest in Ghana. The Journal of Ecology, 64(3), 913. https://doi.org/10.2307/2258816

Matsuo, T., van der Sande, M. T., Amissah, L., Dabo, J., Mohammed Abdul, S., & Poorter, L. (2023). Herbaceous species and dry forest species have more acquisitive leaf traits than woody species and wet forest species. Functional Ecology, 38(1), 194–205. https://doi.org/10.1111/1365-2435.14477
